# BAP31 Promotes Angiogenesis via Galectin-3 Upregulation in Neuroblastoma

**DOI:** 10.3390/ijms25052946

**Published:** 2024-03-03

**Authors:** Mwichie Namusamba, Yufei Wu, Jiaying Yang, Qi Zhang, Changli Wang, Tianyi Wang, Bing Wang

**Affiliations:** College of Life Science and Health, Northeastern University, 195 Chuangxin Road, Hunnan District, Shenyang 110819, China; mwichie.namusamba@unza.zm (M.N.); 2301528@stu.neu.edu.cn (Y.W.); 2201476@stu.neu.edu.cn (J.Y.); aca_zq@163.com (Q.Z.); wchl0918@163.com (C.W.)

**Keywords:** BAP31, Galectin-3, VEGFA, angiogenesis, neuroblastoma, cancer, conditioned media

## Abstract

Neuroblastoma (NB) is one of the highly vascularized childhood solid tumors, and understanding the molecular mechanisms underlying angiogenesis in NB is crucial for developing effective therapeutic strategies. B-cell receptor-associated protein 31 (BAP31) has been implicated in tumor progression, but its role in angiogenesis remains unexplored. This study investigated BAP31 modulation of pro-angiogenic factors in SH-SY5Y NB cells. Through protein overexpression, knockdown, antibody blocking, and quantification experiments, we demonstrated that overexpression of BAP31 led to increased levels of vascular endothelial growth factor A (VEGFA) and Galectin-3 (GAL-3), which are known to promote angiogenesis. Conditioned medium derived from BAP31-overexpressing neuroblastoma cells stimulated migration and tube formation in endothelial cells, indicating its pro-angiogenic properties. Also, we demonstrated that BAP31 enhances capillary tube formation by regulating hypoxia-inducible factor 1 alpha (HIF-1α) and its downstream target, GAL-3. Furthermore, GAL-3 downstream proteins, Jagged 1 and VEGF receptor 2 (VEGFR2), were up-regulated, and blocking GAL-3 partially inhibited the BAP31-induced tube formation. These findings suggest that BAP31 promotes angiogenesis in NB by modulating GAL-3 and VEGF signaling, thereby shaping the tumor microenvironment. This study provides novel insights into the pro-angiogenic role of BAP31 in NB.

## 1. Introduction

Neuroblastoma (NB) is a prevalent pediatric solid tumor originating from neural crest progenitor cells [1]. It ranks as one of the most frequent childhood tumors, comprising approximately 7% of cancer cases in patients under 15 years old and standing as the second most prevalent childhood malignancy [2]. The tumors are primarily located in the abdomen (adrenal medulla) but can also manifest in other areas such as the neck, chest, and pelvis [3]. Neuroblastoma exhibits a wide range of clinical behaviors, with some tumors undergoing spontaneous regression, whereas others follow an aggressive course. Extensive research has been conducted on this childhood cancer, including studies on infant screening, stem cell transplantation, prognostic genes, drug-based treatments, and DNA methylation fingerprints [4,5,6,7,8,9]. 

Angiogenesis is defined as the growth of new blood vessels from preexisting ones and is very important in tumor development. In angiogenesis, several molecular pathways involve transcription factors and proto-oncogenes that support tumor establishment, dissemination, and growth [10]. Neuroblastoma exhibits various mechanisms of angiogenesis, including sprouting from preexisting capillaries, vessel intussusception involving the splitting of existing vessels into two new structures, and vascular mimicry, where malignant cells adopt endothelial-like properties [11]. Angiogenesis predominantly relies on the vascular endothelial growth factor (VEGF) and its corresponding mitogenic receptors found on endothelial cells. In the context of neuroblastoma, the crosstalk between VEGF generated by tumor cells and VEGFR2 plays a pivotal role in facilitating neovascularization [12]. Strategies targeting the VEGF-VEGFR2 signaling pathway have inhibited angiogenesis and slowed tumor growth. For example, melatonin has been found to inhibit angiogenesis in neuroblastoma cells by downregulating VEGF [13]. Another protein involved in angiogenesis is Galectin-3 (GAL-3), which plays a significant role in this process [14]. GAL-3 induces angiogenesis and can be inhibited by competitive saccharides such as lactose and modified citrus pectin (MCP) [15,16,17]. GAL-3 is expressed in most differentiated neuroblastic tumors and is considered an unfavorable biomarker for therapy in high-risk neuroblastomas [18].

B-cell receptor-associated protein 31 (BAP31) is a protein located in the endoplasmic reticulum (ER) that is involved in transporting and regulating molecules related to cell death and cancer progression. BAP31 is linked to cancer cell proliferation, metastasis, and shortened survival [19,20,21]. Recently, BAP31 was reported to promote angiogenesis in colorectal cancer [22]. Due to its widespread expression, BAP31 has emerged as a promising candidate for potential cancer therapy strategies aimed at inducing apoptosis and advancing vaccine development. Notably, BAP31 has been implicated as an unfavorable prognostic marker in head and neck cancer and a potential biomarker for poor prognosis post-surgery in colorectal cancer. In colorectal cancer, downregulation of BAP31 through overexpression of MicroRNA-451a inhibited tumor growth and induced ER stress [23,24].

Furthermore, BAP31 has been shown to regulate hyper-proliferation and metastasis in cervical cancer through its interaction with the HPV16-E5 protein. Targeting BAP31 with MicroRNA-362-3p inhibited cervical cancer cell migration and invasion [25,26,27]. Previous studies have demonstrated that a BAP31 intrabody induced cell death in gastric cancer by inhibiting cyclin-dependent kinase inhibitor 1B (CDKN1B, p27kip1) proteasome degradation [28]. Though BAP31 is linked to cancer cell proliferation, metastasis, and survival, very little research has been conducted about its involvement in angiogenesis. 

This study reports increased tubulogenesis of human umbilical vein endothelial cells (HUVECs) cultured in conditioned media from SH-SY5Y cells overexpressing BAP31. Furthermore, our analysis of the relationship between BAP31 and tumor-associated antigens revealed a positive correlation between BAP31 expression and the levels of GAL-3 and VEGFA. Consequently, our study is the first to report that BAP31 can modulate the expression of pro-angiogenic signaling molecules and regulators in SH-SY5Y cells. Moreover, the secretome from BAP31-overexpressing NB cells exerts a paracrine effect on endothelial cells within the tumor microenvironment, triggering the tubulogenesis of these cells.

## 2. Results

### 2.1. BAP31-Conditioned Media Stimulated HUVEC Cell Migration and Capillary Structure Formation

**Figure 1:**
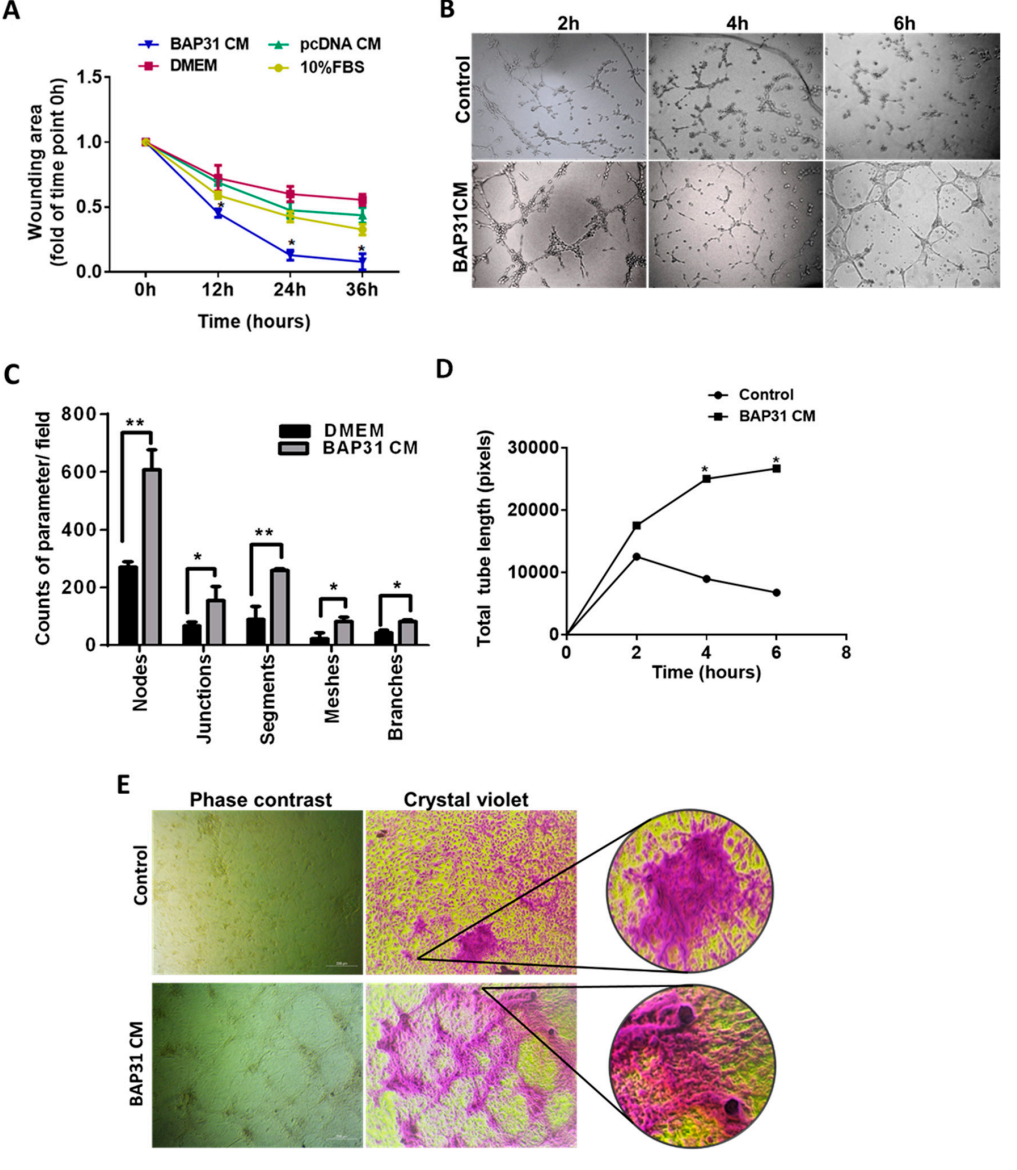
BAP31-conditioned medium stimulates wound healing and microvascular network formation in HUVECs. (**A**) Quantitative analysis of wound closure as a function of time. The rate of wound closure was determined as the wound area at a given time relative to the original wound area. (**B**) Representative photomicrographs of HUVEC capillary tube formation 2 to 6 h after culture in DMEM (control) and BAP31 CM. (**C**) Bar graph demonstrating tube formation parameters. (**D**) Representative graph based on time measurements showing total tube length formation in HUVEC (unit: pixels). (**E**) Tube formation micrographs after 36 h of periodic medium change and 0.1% Crystal Violet staining. Magnification = 5×, scale = 200 µm. Both the wound area and tube networks were measured with ImageJ software (Java 1.8.0), and data are presented as mean values of four independent experiments. Statistical significance was determined by either ANOVA or Student’s *t*-test (* *p* < 0.05 and ** *p* < 0.01).

The conditioned medium (CM) from SH-SY5Y cells overexpressing BAP31 was used to study the migratory behavior of endothelial cells in a wound-healing assay. The results demonstrated that HUVECs treated with BAP31 CM exhibited significantly accelerated wound coverage compared to controls over a 36 h period (Figure S1). The wound coverage rates were quantified, showing that BAP31 CM increased the migration of HUVECs by 90%, 50%, and 10% faster than treatments with DMEM, pcDNA CM, and 10% FBS, respectively (Figure 1A). Furthermore, we explored the impact of BAP31 CM on endothelial tube formation on Matrigel. The results indicated that BAP31 CM significantly enhanced tube formation compared to the control medium. Parameters such as nodes, segments, meshes, and junctions were analyzed to characterize the network formation (Figure 1B,C). Notably, the length of tubes formed in BAP31 CM was significantly longer than those in the control medium (*p* < 0.05). Tube formation was initiated within two hours of culture. However, there was a decrease in tube length after six hours (Figure 1D).

Media replenishing every six hours over a 36 h cell culture period sustained tube formation and elongation (Figure 1E). Crystal Violet staining confirmed that BAP31 CM induced the formation of closed loops by HUVECs. Conversely, cells cultured in the control medium formed clusters and eventually underwent cell death. Markedly, when magnified (5×), structures at internodes in BAP31 CM displayed tubular-like cavities, adding an interesting morphological dimension to the observed effects. 

Moreover, SH-SY5Y cells overexpressing BAP31 exhibited tube formation on Matrigel, a process known as vascular mimicry, and BAP31 knockdown (siBAP31) inhibited this process. As shown in Figure S2, after 24 h of cell culture, the tube formation parameters in SH-SY5Y cells overexpressing BAP31 were systematically higher in counts of nodes, junctions, meshes, segments, and branches compared to the control. Meanwhile, BAP31 knockdown reduced the counts of nodes, meshes, segments, and branches, with an insignificant difference in junctions compared to the control. Generally, these findings suggest that BAP31 promotes endothelial cell migration, accelerates wound closure, enhances tube formation, and influences the morphological characteristics of the formed structures, indicating a potential role for BAP31 in angiogenesis and vascular remodeling.

### 2.2. The Relationship of BAP31 with Pro-Angiogenic Factors

To investigate the relationship between BAP31 and pro-angiogenic factors, we utilized the Proteome Profiler Human XL Oncology Array (antibody chip) comprising 84 cancer-related antigens. By overexpressing BAP31 in SH-SY5Y cells, we analyzed the cell lysate and identified several molecules associated with angiogenesis regulation. Among these molecules, 15 were selected for further analysis based on their fold change upon BAP31 overexpression (Figure S3). Notably, pro-angiogenic factors such as Gelsolin-like capping protein (Cap G), Galectin-3 (LGALS3, GAL-3), matrix metalloproteinases 1 and 2 (MMP-2 and MMP-3), VEGFA, Cathepsin S (CTSS), Vimentin (VIM), Osteopontin (SPP1), fibroblast growth factor (FGF), platelet-derived growth factor (PDGFAA), and Survivin (BIRC5) were up-regulated. In contrast, Delta-Like canonical Notch ligand 1 (DLL1) remained unchanged. Interleukin 6 (IL-6), Mesothelin (MSLN), and secreted protein acidic and cysteine-rich (SPARC) proteins were down-regulated.

**Figure 2:**
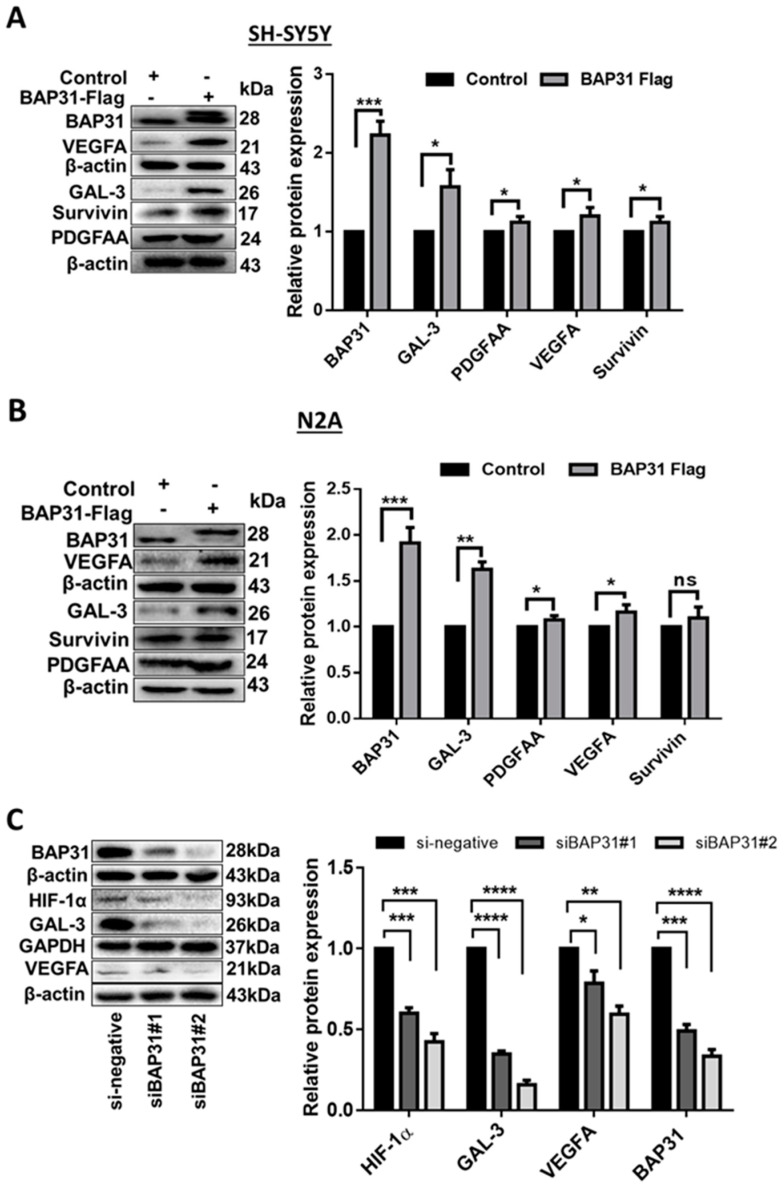
Effect of BAP31 overexpression and knockdown on angiogenic factors. Western blots and bar graphs of BAP31 overexpression in (**A**) SH-SY5Y cells and (**B**) N2A cells showing up-regulation of VEGFA, GAL-3, PDGFAA, and Survivin. (**C**) Western blots and bar graphs of BAP31 knockdown showing down-regulation of GAL-3, VEGFA, and HIF-1α. The bar graphs were obtained by normalizing to β-actin (or GAPDH). Each bar represents the means ± S.E.M. of three independent experiments, and statistical significance was determined by Student’s *t*-test (* *p* < 0.05, ** *p* < 0.01, *** *p* < 0.001, and **** *p* < 0.0001; ns, non-significant).

Known pro-angiogenic factors, including GAL-3, VEGFA, Survivin, and PDGFAA, exhibited a fold difference of 1.8, 2.1, 1.9, and 2.1, respectively. To validate these findings, we examined their protein levels by Western blotting in SH-SY5Y and N2A cells overexpressing BAP31 (Figure 2). The protein expression changes for all four proteins were significant and consistent with the antibody chip detection in SHSY-5Y cells but not in N2A cells. In SH-SY5Y-BAP31 cells, BAP31 increased by 129% (*p* < 0.0001), GAL-3 by 90% (*p* < 0.01), PDGFAA by 33% (*p* < 0.01), VEGFA by 120% (*p* < 0.001), and Survivin by 42% (*p* < 0.01). Similarly, in N2A-BAP31 cells, BAP31 increased by 104% (*p* < 0.001), GAL-3 by 93% (*p* < 0.01), and VEGFA by 92% (*p* < 0.001) compared to the control. Although the upregulation of PDGFAA by 15% (*p* < 0.05) was significant, it was inconsistent with the antibody chip result. In addition, the upregulation of Survivin by 12% (*p* = 0.0919) was statistically insignificant compared to the control, as shown in Figure 2B. 

Based on these observations, we embarked on investigating the effect of BAP31 knockdown on GAL-3 and VEGFA in SH-SY5Y cells. Also, given that HIF-1α is a regulator of GAL-3 and VEGFA, we examined the levels of HIF-1α in cells with BAP31 knockdown. Western blot analysis confirmed the successful knockdown of BAP31 (Figure 2C), with siBAP31#1 showing a 49% reduction (*p* < 0.01) and siBAP31#2 showing a 65% reduction (*p* < 0.001) in protein levels compared to the control (si-negative) group (Figure 2C). Interestingly, the decrease in BAP31 expression correlated with a significant reduction in GAL-3 levels by 65% (*p* < 0.0001) for siBAP31#1 and 84% (*p* < 0.0001) for siBAP31#2. Similarly, VEGFA levels were significantly decreased by 22% (*p* < 0.05) for siBAP31#1 and 41% (*p* < 0.01) for siBAP31#2. Furthermore, the expression of HIF-1α was significantly reduced by 40% and 56% (*p* < 0.001) in siBAP31#1 and siBAP31#2, respectively. These findings demonstrate a positive correlation between BAP31 and GAL-3 and VEGFA expression. Additionally, they suggest a potential role for BAP31 to regulate the expression of HIF-1α.

### 2.3. BAP31 Regulates GAL-3 and VEGFA by Targeting HIF-1α in SH-SY5Y Cells

**Figure 3:**
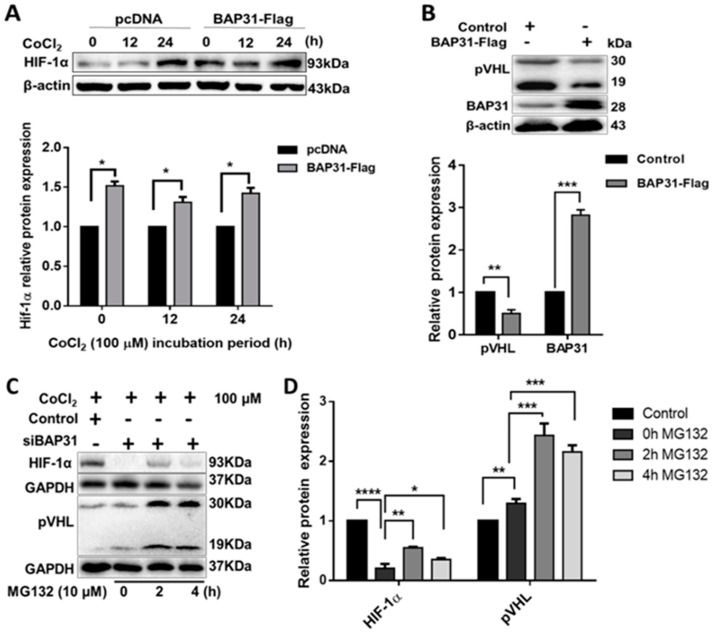
BAP31 augments hypoxia-induced HIF-1α stability. (**A**) Western blots and bar graph of HIF-1α protein expression in SH-SY5Y-BAP31 and control cells at 0, 12, and 24 h exposure to CoCl_2_. (**B**) Western blots and bar graphs of pVHL downregulation in SH-SY5Y-BAP31 and control cells. (**C**) Western blots and (**D**) bar graphs of HIF-1α and pVHL expression determined in hypoxia-like conditions by treatment with CoCl_2_ (100 µM) followed by treatment with MG132 (10 µM) in SY-SY5Y BAP31 knockdown. The bar graphs were obtained by normalizing to β-actin or GAPDH. Each bar represents the means ± S.E.M. of three independent experiments, and statistical significance was determined by ANOVA (* *p* < 0.05) and a Student’s *t*-test (* *p* < 0.05, ** *p* < 0.01, *** *p* < 0.001, and **** *p* < 0.0001).

The tumor microenvironment characterized by low oxygen levels is crucial in stimulating angiogenesis in surrounding stromal and endothelial cells. Neuroblastoma has notable differential expression of the oxygen-sensitive HIFα subunits (HIF-1α and HIF-2α) at the protein level [29]. Elevated levels of hypoxia-inducing factors contribute to the secretion of various pro-angiogenic factors, including VEGFA, PDGF, MMPs, and integrins. To mimic hypoxic conditions, SH-SY5Y cells were treated with 100 µM of CoCl_2_×6H_2_O (CoCl_2_), and HIF-1α protein expression was assessed from cell lysates. Time-dependent analysis revealed that HIF-1α expression increased steadily in both SH-SY5Y-BAP31 cells and the control (pcDNA) from 0 to 24 h following CoCl_2_ treatment (Figure 3A). Markedly, BAP31 overexpression resulted in higher levels of HIF-1α compared to the control. Specifically, there was a 7% increase from 0 to 12 h and a 39% increase from 12 to 24 h, resulting in an overall increase of 46% (*p* = 0.0047) from 0 to 24 h under hypoxic conditions. These findings highlight the ability of BAP31 to upsurge the effects of hypoxia in tumors, including the regulation of HIFα subunits and their associated regulatory factors, such as von Hippel–Lindau protein (pVHL). 

The pVHL protein is a known regulator of HIF-1α degradation through ubiquitination [30]. Our study observed a 50% reduction in pVHL expression (*p* = 0.0044) upon overexpression of BAP31 (Figure 3B). This finding led us to investigate whether BAP31 knockdown would increase pVHL expression. Considering the susceptibility of HIF-1α to proteasome-mediated degradation, we utilized the proteasome inhibitor MG132 to prevent its degradation in siBAP31 cells and si-negative control cells. Interestingly, even with the stabilization of HIF-1α by MG132, its expression was still downregulated in siBAP31 cells, which inversely correlated with the upregulation of pVHL (Figure 3C,D). Following treatment with MG132 for 2 h and 4 h, HIF-1α expression significantly increased in siBAP31 cells compared to the control at 0 h (*p* < 0.001 and *p* < 0.05, respectively). Concurrently, there was a significant increase in pVHL protein expression between si-negative and siBAP31 cells at 0 h post-MG132 treatment (*p* < 0.01). Furthermore, both isoforms of pVHL, namely, pVHL30 and pVHL19, were detected. Consistent with our previous findings (Figure 2C), these data further support the potential regulatory role of BAP31 in modulating the stability and expression of HIF-1α through its influence on pVHL levels.

### 2.4. Hypoxic BAP31-Conditioned Media Up-Regulates HIF-1α, VEGFA, and VEGFR2 in HUVECs

**Figure 4:**
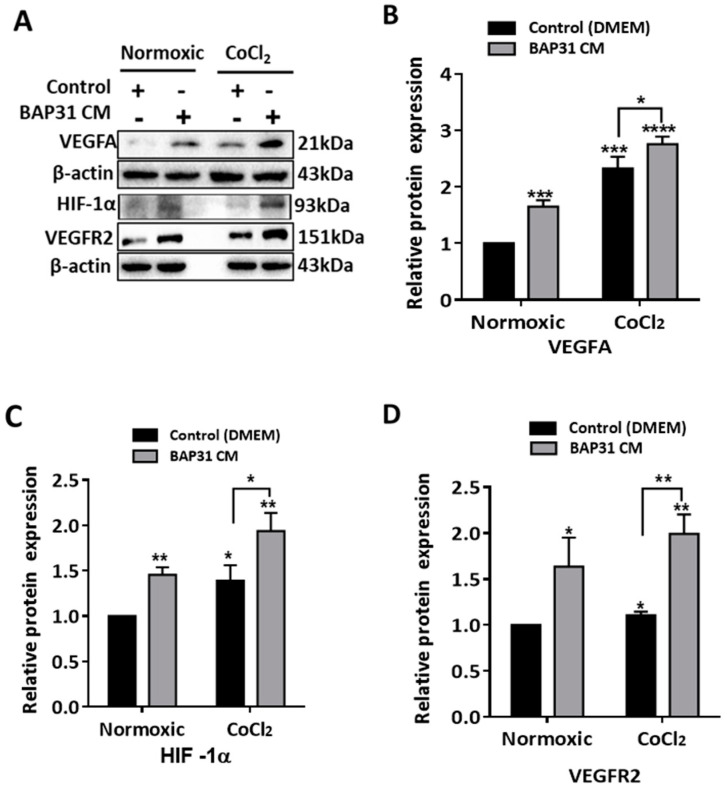
Effect of normoxic and hypoxic BAP31-conditioned media on pro-angiogenic factors in HUVECs. (**A**) Western blots of cell lysate after 24 h of culture in CoCl_2_ BAP31 CM and control (pcDNA CM). Bar graphs of significant difference in (**B**) VEGFA (*p* < 0.001), (**C**) HIF-1α (*p* < 0.01), and (**D**) VEGFR2 (*p* < 0.01) expression between the normoxic control and CoCl_2_ BAP31 CM. The bar graphs were obtained by normalizing to β-actin. Each bar represents the means ± S.E.M. of three independent experiments. Statistical significance was determined by a Student’s *t*-test (* *p* < 0.05, ** *p* < 0.01, *** *p* < 0.001, and **** *p* < 0.0001).

Given the observed increase in HIF-1α expression when SH-SY5Y cells were treated with CoCl_2_, we explored the impact of hypoxic conditioned media from BAP31-overexpressing SH-SY5Y cells on HUVECs. We assessed HIF-1α, VEGFA, and VEGFR2 protein expression levels in HUVECs cultured in normoxic and CoCl_2_—induced hypoxic BAP31-conditioned media. Western blot analysis revealed interesting findings, as shown in Figure 4A. VEGFA expression was significantly up-regulated in normoxic BAP31 CM (*p* < 0.001) when compared to the control (DMEM), as shown in Figure 4B. Under normoxic conditions, VEGFA expression may have a baseline level or could be influenced by factors in the BAP31 CM. In CoCl_2_—induced hypoxia, the presence of BAP31 CM still allowed for the upregulation of VEGFA in CoCl_2_—control (*p* < 0.001) and CoCl_2_—BAP31 CM (*p* < 0.0001) when compared to the normoxic control, suggesting that factors contending in BAP31 CM may contribute to maintaining or enhancing VEGFA expression under hypoxic conditions. HIF-1α expression levels were up-regulated under normoxic conditions (*p* < 0.05), and compared to the control (normoxic), CoCl_2_-induced hypoxia also exhibited elevated HIF-1α expression: (45%, *p* < 0.05) and (94%, *p* < 0.01) for CoCl_2_ (control) and CoCl_2_—BAP31 CM, respectively. The upregulation of HIF-1α in HUVECs treated with normoxic BAP31 CM could be attributed to factors in the conditioned media that may induce a response similar to hypoxia, leading to HIF-1α stabilization. In addition, its protein expression was significantly higher under hypoxia (*p* < 0.05), with a notable difference between the two conditions (*p* < 0.001) (Figure 4C). 

Considering the alterations in VEGFA expression, we investigated the primary receptor of VEGFA, VEGFR2, to assess the paracrine effect. VEGFR2 exhibited upregulation in response to BAP31-conditioned media (CM) under both normoxic and hypoxic conditions (*p* < 0.05). Remarkably, its expression in BAP31 CM was higher under hypoxic conditions (*p* < 0.01) compared to normoxic conditions (*p* < 0.05) (Figure 4D). These findings further support our previous hypothesis that BAP31 plays a role in influencing HIF expression even under normoxic conditions and enhances the effects of hypoxia in cancer. Moreover, the increased expression of VEGFR2 suggests external signaling from the BAP31 CM.

Furthermore, substantial evidence supports the involvement of VEGFA/VEGFR2 autocrine and paracrine responses in endothelial and tumor cells during tumor progression. For instance, a protective role of the VEGFA/VEGFR2 autocrine loop was reported in epithelial ovarian carcinoma cells, preventing them from undergoing anoikis-induced cell death. Additionally, autocrine loop mechanisms involving VEGF/VEGFR2 have been reported to regulate the survival of specific subsets of acute leukemia [31,32].

### 2.5. Antibody Blocking of GAL-3 Reduced Angiogenic Ability and Downregulated GAL-3 Downstream Target Molecules in Endothelial Cells

**Figure 5:**
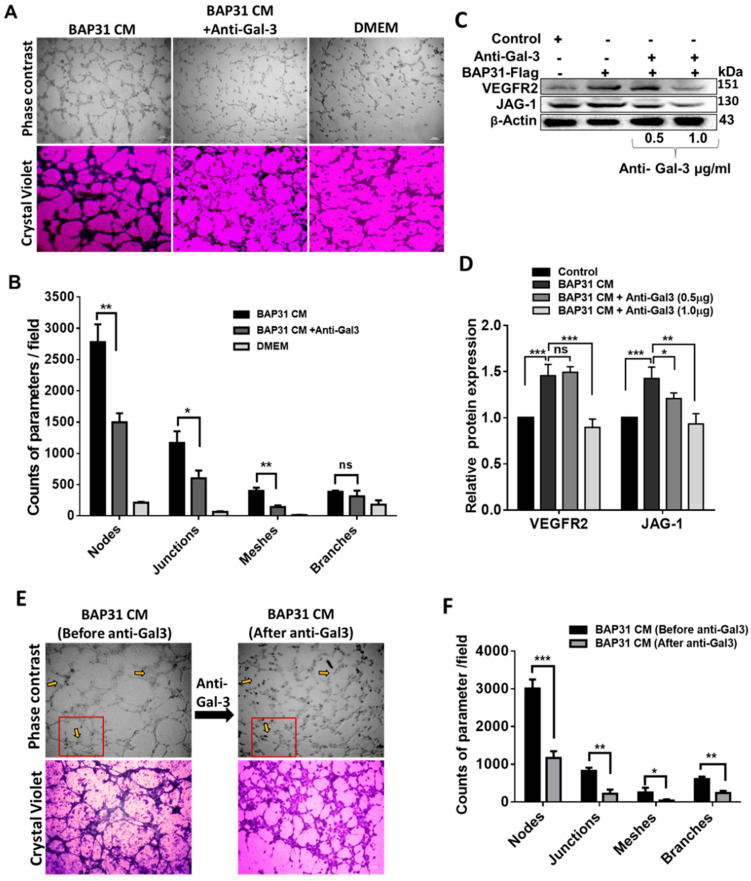
Suppression of microvascular network formation and downregulation of Galectin 3 downstream targets in HUVECs. (**A**) Photomicrographs depicting diminished tube formation in BAP31 CM containing anti-Gal 3; scale = 200 µm. (**B**) Bar graph representing tube formation parameter difference, indicating the number of nodes (*p* < 0.01), junctions (*p* < 0.05), meshes (*p* < 0.01), and branches (*p* = 0.262) between BAP31 CM and anti-Gal3. (**C**) Western blot analysis showing the impact of anti-Gal 3 on Galectin-3 downstream targets. (**D**) Bar graphs representing protein expression level changes in Galectin-3 targets; VEGFR2 and JAG-1 were significantly down-regulated at higher anti-Gal 3 treatment of 1.0 µg/mL. The bar graphs were obtained by normalizing to β-actin, and each bar represents the means ± S.E.M. of three independent experiments. (**E**) Photomicrographs of tube formation in BAP31 CM before and after addition of anti-Gal 3. (**F**) Bar graph showing the effect of anti-Gal 3 on established tube formation parameters in BAP31 CM. Each bar represents the means ± S.E.M. of five independent experiments. Statistical significance was determined by a Student’s *t*-test (* *p* < 0.05, ** *p* < 0.01, *** *p* < 0.001; ns, non-significant).

Competitive blocking of GAL-3 has been shown to down-regulate intrinsic GAL-3 expression, reducing cell morphogenesis and angiogenic capability in endothelial cells [15]. To further explore the impact of BAP31-mediated augmentation of GAL-3 on angiogenesis, we blocked GAL-3 with a primary antibody in HUVECs and studied the changes in the tube structures (Figure 5A,B). In the presence of BAP31-anti-Gal 3−conditioned media, there was a significant reduction in nodes (*p* = 0.0088), junctions (*p* = 0.0117), and meshes (*p* = 0.0063), indicating a decrease in angiogenic capability. However, there was no significant difference in the number of branches (*p* = 0.2625) compared to the control.

In the context of endothelial cell-surface angiogenic receptors, a pronounced elevation in VEGFR2 expression (45%, *p* < 0.001) was observed in HUVECs treated with BAP31 CM compared to the control. Notably, at a lower concentration of anti-Gal 3 (0.5 µg/mL), VEGFR2 expression was insignificantly altered (4%, *p* = 0.6033). At a higher concentration (1.0 µg/mL), anti-Gal 3 demonstrated a notable inhibitory effect on VEGFR2 expression, resulting in a statistically significant reduction of 56% compared to the control group (*p* < 0.001), substantiating the regulatory influence of GAL-3 in modulating VEGFR2 expression. In addition, Jagged1 (JAG-1), a pivotal player in tip cell-stalk cell Notch signaling known to uphold low VEGFR expression levels in stalk cells while fostering stalk cell proliferation through sensitivity to VEGFA [33], exhibited heightened expression (*p* < 0.001) in HUVECs subjected to BAP31 CM compared to the control. Intriguingly, in the presence of anti-Gal 3 at 0.5 and 1.0 µg/mL, JAG-1 expression was significantly down-regulated by 22% (*p* < 0.05) and 49% (*p* < 0.01), respectively.

Furthermore, endothelial cells manifesting angiogenic morphologies lost their pro-angiogenic capacity upon exposure to anti-Gal 3 (Figure 5E,F). The diminished pro-angiogenic phenotype was validated by reduced counts of key angiogenic parameters, including nodes (*p* < 0.001), junctions (*p* < 0.01), meshes (*p* < 0.05), and branches (*p* < 0.01), providing additional support for the pro-angiogenic role of BAP31 through the upregulation of GAL-3. These findings align with previous investigations positing GAL-3 as a molecular regulator of the JAG-1/Notch-1 signaling pathway. Specifically, GAL-3 secretion by cancer cells has been implicated in enhancing sprouting angiogenesis via the JAG-1 ligand [34]. Consequently, the results of our study elucidate the fact that the BAP31-induced upregulation of GAL-3 expression in cancer cells, along with its subsequent secretion into the conditioned media, is sufficient to initiate JAG1—regulated endothelial cell morphogenesis towards angiogenic phenotypes.

## 3. Discussion

This study demonstrates an unidentified function of BAP31 protein to promote angiogenesis in neuroblastoma (NB), as shown in Figure 1, where conditioned media from cancer cells overexpressing BAP31 induced in vitro tubulogenesis of endothelial cells. BAP31 overexpression in SH-SY5Y and N2A NB cell lines up-regulated many pro-angiogenic cytokines, including GAL-3 and VEGFA (Figure 2). In neuroblastoma, cancer cells secrete VEGFA, which is involved in the VEGFA/VEGFR2 signaling pathway and promotes vascularization, into the microenvironment [13]. In addition, VEGFR2 endothelial cell-surface expression and activation are mediated by GAL-3 [35]. In our study, both VEGFA and GAL-3 positively correlated with BAP31 overexpression and knockdown in SH-SY5Y neuroblastoma cells. Furthermore, under hypoxic conditions, HIF-1α acts as a transcription factor, orchestrating the response to oxygen deprivation by stimulating the expression of GAL-3 and VEGFA [36,37]. HIF-1α positively correlated with BAP31 overexpression, and the knockdown of BAP31 reduced the expression of HIF-1α despite protein stabilization with MG132 (Figure 3). In a recent study, BAP31 knockdown positively correlated with GAL-3 in colorectal cancer and affected cancer cell stemness [22]. These findings underscore the role of BAP31 in mediating the expression of GAL-3 and VEGFA in neuroblastoma.

pVHL is a negative modulator of HIF-1α activity, and a reduction in pVHL can increase the level of HIF-1α. BAP31 overexpression down-regulated pVHL protein levels, but in BAP31 knockdown in combination with CoCl_2_ and MG132 treatments, pVHL expression was up-regulated and inversely correlated with HIF-1α (Figure 3). In the cancer cell, pVHL acts as a target recruitment subunit in the E3 ubiquitin ligase complex and recruits hydroxylated HIF-1α under normoxic conditions. Loss of pVHL expression due to gene deletion, promoter hyper-methylation, or mutations affects its ability to regulate HIF-1α, resulting in constitutive HIF stabilization and activation, irrespective of oxygen levels. It is important to note that the pVHL gene is not mutated in neuroblastoma cell lines [38]. BAP31 targets the ubiquitin-tagged proteins for degradation by the proteasome in the cytoplasm. However, there is no report on whether it is involved in the degradation process of HIF-1α. Based on our findings, we suspect that BAP31 interacts with pVHL in the ER, and that BAP31 overexpression in NB downregulates pVHL, impeding its regulatory role on HIF-1α. pVHL may still bind to HIF-1α, but at low expression levels, there is more unbound HIF-1α in the cell, subsequently increasing GAL-3 and VEGFA expression [30].

Here, we showed that CoCl_2_ treatment increased HIF-1α in both pcDNA (control) and BAP31-overexpressing cells (Figure 3A). In addition, VEGFR2, which is a GAL-3 and VEGFA target, was markedly up-regulated in both normoxic and CoCl_2_ media in HUVECs (Figure 4). Since neuroblastomas are highly hypoxic, these data suggest that BAP31 enhances the hypoxic and nutrient deprivation-dependent GAL-3 and VEGFA up-regulation. Furthermore, the inverse correlation between pVHL and HIF-1α has been reported many times, and the mechanism is well established. However, for the first time, we relate the positive correlation between BAP31 and HIF-1α in cancer, specifically NB.

In recent years, antitumor therapies that block VEGF and VEGFRs to prevent or recede tumor vascularization have shown much promise in many cancers, including breast and hepatocellular carcinoma [39,40]. Our study showed that blocking GAL-3 with antibodies (Figure 5) reduced the pro-angiogenic ability of HUVECs and downregulated GAL-3 downstream target molecules, including VEGFR2 and JAG-1. Moreover, low expression of GAL-3 is a favorable prognostic factor in stomach and colorectal cancer [41]. We thus reasoned that BAP31 modulates angiogenesis by influencing the pVHL/HIF-1α axis, which up-regulates the expression of GAL-3 and, consequently, increases secreted GAL-3 and VEGFA and the promotion of angiogenesis by endothelial cells. This intricate interplay between BAP31, GAL-3, and VEGFA implies that VEGFA might be a prerequisite for GAL-3 function in NB angiogenesis. Together, these molecules are secreted into the microenvironment by tumor cells, where they exert their angiogenic capabilities. GAL-3, through its regulatory influence on endothelial cell morphological changes in angiogenesis, accentuates Jagged1/Notch signaling. Binding preferentially to Notch ligands, GAL-3 enhances JAG-1 protein half-life over DLL4, thereby activating JAG-1/Notch-1 signaling in endothelial cells [34]. As demonstrated in our research, the conditioned media from SH-SY5Y-BAP31 cells promoted cell migration, induced changes in cell morphology, and stimulated capillary tube formation in HUVECs (Figure 1). Our findings substantiate the fact that BAP31 overexpression significantly influences angiogenesis through GAL-3-mediated signaling in the cancer cell. Moreover, BAP31 overexpression by cancers may represent an alternative mechanism for vascularization in nutrient-starved tumors, facilitating nutrient acquisition for survival and circumventing VEGF/VEGFR anti-angiogenic targeted therapies. In the unfavorable condition of VEGF inhibition, high GAL-3 expression serves as a stimulant for vascularization through alternative pathways independent of the VEGF/VEGFR2 signaling pathway [42,43].

**Figure 6:**
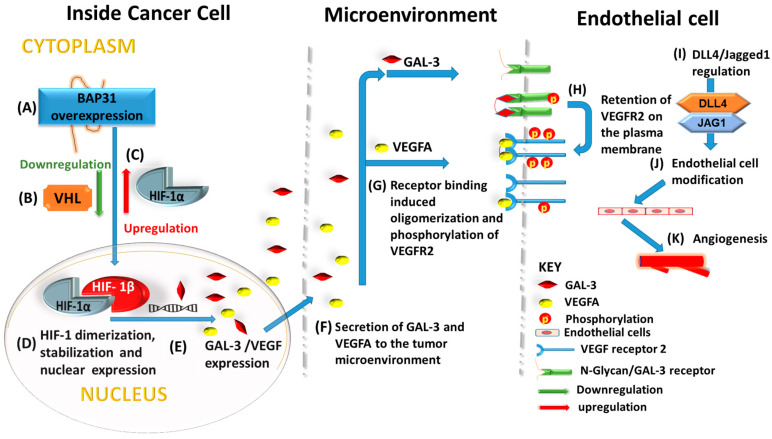
Schematic mechanism of BAP31 regulation of angiogenesis in neuroblastoma. Inside the cancer cell, (A) BAP31 overexpression induces (B) down-regulation of pVHL, which consequently (C) increases HIF-1α expression and nuclear transport. (D) HIF-subunits dimerize, (E) regulating GAL-3 and VEGFA expression. (F) Secretion of GAL-3 and VEGFA into the tumor microenvironment results into (G) VEGFR and N-Glycan receptor binding by VEGFA and GAL-3 and subsequently induces phosphorylation of VEGFR2. GAL-3 regulates (H) the retention of VEGFR2 on the plasma membrane and regulation of (I) the determinants of endothelial cell tip-cell/stalk-cell modification (Dll4 and Jagged1). In response (J), endothelial cells undergo proangiogenic modification leading to (K) vessel formation.

Though the molecular mechanism underlying BAP31’s regulation of angiogenesis remains unknown, our study, coupled with evidence implicating BAP31 in various facets of cancer cell biology, including proliferation, migration, metastasis, and survival, allows us to propose a model (Figure 6). In this model, BAP31 does not act in isolation but functions as a collaborative partner or complex with other regulatory molecules. Given the indispensability of angiogenesis for tumor growth, it is conceivable that increased BAP31 expression up-regulates GAL-3, thereby intensifying tumorigenesis by potentiating VEGFR2 signaling pathways. This, in turn, enhances the sensitization of endothelial cells to VEGFA stimuli, ultimately promoting angiogenesis.

## 4. Materials and Methods

### 4.1. Cell Culture

The human neuroblastoma cell lines SH-SY5Y and N2A and human umbilical vein endothelial cells (HUVECs) were obtained from the American Type Culture Collection (ATCC). The cell lines were cultured in Dulbecco’s Modified Eagle Medium (DMEM, Gibco, New York, NY, USA), supplemented with 10% fetal bovine serum (FBS), 1% L-glutamine, and 1% penicillin-streptomycin (Beyotime, Shanghai, China), and incubated in a 5% CO_2_ atmosphere at 37 °C.

### 4.2. Plasmid Transfection

The pcDNA3.1(−)BAP31-Flag plasmids are maintained in our laboratory and were constructed as previously described [44]. Briefly, plasmids were constructed from full-length human *BCAP31* cDNA derived from HeLa cell mRNA. *BCAP31* cDNA was ligated to a pcDNA3.1 (−)vector with a Flag tag by PCR. The pcDNA3.1 (−) vector without the *BCAP31* insert was used as a control. The plasmids were confirmed by sequencing before use (Genewiz Biotechnology Co., Ltd., Suzhou, China). SH-SY5Y and N2A cells were transiently transfected using Lipofectamine^®^ 2000 reagent (Invitrogen, Carlsbad, CA, USA). Briefly, 5 × 10^5^ cells were seeded in 6-well plates the day before transfection. A plasmid-to-Lipofectamine ratio of 1:1 was used. The total plasmids were mixed with 500 µL of Opti-MEM for 5 min; simultaneously, the Lipofectamine 2000 reagent was diluted into 500 µL of Opti-MEM for 5 min. Then, the diluted Lipofectamine 2000 was added to the DNA complex. The solution was mixed gently and incubated at room temperature for 20 min. Next, the solutions were added to the cells drop by drop. After six hours, the medium was replaced with fresh medium, and the cells were harvested 48 h later.

### 4.3. RNA Interference of BAP31

The short interfering RNA (siRNA) targeting BAP31 and the scrambled non-target negative control were obtained from GenePharma (Shanghai, China). The designed siRNA sequences targeted the coding region of BAP31 [44], including BAP31–siRNA-1 (5′-CUCAGAGGAAUCUCUAUAUTT-3′), BAP31–siRNA-2 (5′-GAGGGCCUUACCAAAGAAUTT-3′), and BAP31–siRNA-3 (5′-CCUCCAAUGAAGCCUUUAATT-3′). A scrambled RNA sequence (5′-TTCTCCGAACGTGTCACGUTT-3′) served as a mock transfection control. The cells were grown to 30–50% confluence and then treated with siRNA transfections at a final concentration of 100 nM. The cells were transfected using Lipofectamine RNAiMax reagents (Invitrogen, Carlsbad, CA, USA) and were harvested after 72 h. The efficiency of BAP31 knockdown was evaluated through Western blot analysis. 

### 4.4. Conditioned Medium Preparation

SH-SY5Y cells overexpressing BAP31 were grown in 100 mm plates to 30–40% confluence in complete media. The cells were washed once with PBS, and the growth medium was replaced with 10 mL of serum-free DMEM for 24 h. The conditioned medium (CM) was harvested when cells reached 80% confluence. The CM was centrifuged to remove cell debris and used immediately or stored at −80 °C. 

### 4.5. Wound-Healing Assay

Control and test group HUVEC cells were seeded in a 24-well plate at 1 × 10^6^ cells per well and cultured as a monolayer overnight to reach confluence. A wound area of about 0.7 mm wide was created by scratching the monolayer with a 100 μL pipette tip, and the cells were washed twice with PBS to remove floating cells. To analyze the effect of BAP31 on HUVE cell migration, the cells were treated with different media: DMEM, pcDNA CM, BAP31 CM, and 10% FBS. Wound closure was monitored at 0, 12, 24, and 36 h. Four images of the wounded cell monolayer were captured at respective times and analyzed with ImageJ software (Java 1.8.0, NIH, Washington, DC, USA). Cell migration activity was expressed as the fold change in the wound area relative to the original wound area immediately after the scratch at 0 h. 

### 4.6. Endothelial Cell Capillary-like Tube Formation Assay

Matrigel thawed at 4 °C was added to the wells of a 96-well microtiter plate (50 μL/well) and allowed to gel at 37 °C for 1 h. HUVECs (1 × 10^5^ per well) were seeded in DMEM supplemented with 1% FBS or BAP31 CM alone. After 2 h, 4 h, and 6 h of incubation, four random fields were selected per well, and tubular structures were photographed and analyzed. Fresh medium was added every 6 h for 36 h. The medium was carefully removed from the wells, and the cells were washed with 50 μL PBS per well. The PBS was carefully removed, and 50 μL of 0.1% of Crystal Violet solution in distilled water (*w*/*v*) was added per well. The plates were incubated for 15 min at room temperature and then washed twice with non-flowing water. An inverted microscope (Leica, Wetzlar, Germany) was used to observe the tube structures. As previously described, the tubular network formation was assessed using the Angiogenesis Analyzer plugin in ImageJ software (Java 1.8.0, NIH, Washington, DC, USA) [45]. The total tube length was determined by the number of branches and segment length per image (units: pixel). 

### 4.7. Human XL Oncology Array Kit

SH-SY5Y cells were transfected with BAP31 or mock-transfected for 48 h. The cells were then collected and analyzed using a Human XL Oncology Array (R&D Systems, Minneapolis, MN, USA), according to the manufacturer’s instructions. Array images were captured and analyzed.

### 4.8. Western Blot Analysis

Cells were lysed in RIPA buffer (Thermo Fisher Scientific, Waltham, MA, USA) enhanced with a 1% protease inhibitor cocktail (Sigma-Aldrich, St. Louis, MO, USA) for 10 min on ice. This was followed by centrifugation at 12,000× *g* for 15 min at 4 °C. Part of the supernatant’s protein concentration was then determined using the BCA protein assay kit from Beyotime (Shanghai, China) with a plate reader (BioTek, VT, USA) at 562 nm. A total of 5 × SDS buffer was added to the remaining supernatant and then boiled for 10 min. Proteins in equal quantities (30–50 µg) were then separated by 8–10% SDS-PAGE and transferred onto a 0.45 µM polyvinylidene fluoride (PVDF) membrane (Merck Millipore, Darmstadt, Germany). Membrane blocking was performed for one hour at room temperature with skimmed milk (5%), followed by incubation with primary antibodies at 4 °C overnight. The membranes were washed three times with Tris-buffered saline containing 0.1% Tween 20 (TBST) and incubated with corresponding secondary antibodies for one hour at room temperature. Protein bands were visualized with an ECL detection kit (Thermo Fisher Scientific) with Bio-Rad ChemiDocTM imaging systems (Bio-Rad, Hercules, CA, USA). The membrane was then stripped and labeled with β-actin (or GAPDH) as per standard Western blot procedures. The intensities of the bands targeted to the proteins were calculated and normalized to that of β-actin (or GAPDH) using Image Lab software (5.2.1, Bio-Rad, Hercules, CA, USA). Three experiments were repeated. The antibodies used in this study were as follows: anti-Galectin 3, anti-VEGFA, anti-HIF1A, anti-VHL, anti-Jagged1, anti-VEGFR2, and anti-GAPDH were all obtained from Wanlei Biotechnology Co., Ltd., Shenyang, China; rabbit anti-BCAP31 (Proteintech, Wuhan, China), chicken anti-BCAP31 (Abcam, Cambridge, UK), mouse anti-β-actin (Abcam, Cambridge, UK), and the secondary antibodies (anti-rabbit IgG and anti-mouse IgG) were obtained from Thermo Fisher Scientific (Waltham, MA, USA).

### 4.9. Proteasome Inhibition (MG132) Assay

The proteasome inhibitor MG132 solution was prepared according to the manufacturer’s manual. A concentration of 10 µM was added to the cells at different time intervals of 0 h (control), 0.5 h, 2 h, 4 h, and 8 h to inhibit protein degradation. Western blotting was then used to determine the expression levels of the protein of interest.

### 4.10. Cobalt (II) Chloride Hexahydrate (CoCl_2_) Assays

Cobalt (II) chloride (CoCl_2_) was used to simulate a hypoxic condition. A stock solution of 25 mM was prepared in 100 mL sterile water. The working solution was determined at different concentration ranges from 25 to 500 µM, with 0 µM (no treatment) as a control in SH-SY5Y cells and HUVECs. An optimum concentration of 100 µM was identified and was thus used in all other experiments. Western blotting was then used to determine the expression levels of the proteins of interest.

### 4.11. Effect of Galectin 3-Neutralizing Antibody on Endothelial Cell Capillary Tube Formation Assay

Matrigel thawed at 4 °C was added to the wells of a 96-well microtiter plate (50 μL/well) and allowed to gel at 37 °C for 1 h. HUVECs (1 × 10^5^ per well) were seeded in BAP31 CM alone or in BAP31 CM supplemented with anti-Gal3 (1.0 µg/mL). DMEM was used as a negative control. The tubes were developed for 36 h (with 6 h periodic media change) in the three different media. Images were captured at 36 h, followed by washing with 50 μL PBS per well. The PBS was carefully removed, and 50 μL of 0.1% of Crystal Violet solution in distilled water (*w*/*v*) was added per well. The plates were incubated for 15 min at room temperature and then washed twice with non-flowing water. The tube structures were observed with an inverted microscope. In another set of experiments, BAP31 CM was used to develop the tubes for 18 h. At the third media change, Galectin 3-neutralizing antibody was added, and the tubes were observed. Images were taken at 24 h, and corresponding 18 h and 24 h wells were stained with Crystal Violet, as explained above. ImageJ software (Java1.8.0, NIH, Washington, DC, USA) analyzed tubular network formation. 

### 4.12. Statistical Analysis

All experiments were performed in triplicate, and the data shown represent consistently observed results. Statistical analyses were performed using ImageJ (Java1.8.0, NIH, Washington, DC, USA) and GraphPad Prism 5.0 (GraphPad, La Jolla, CA, USA) software. Data were analyzed using the Student’s *t*-test, or analysis of variance, as appropriate to the experiment or context. Statistical significance was expressed as *, **, ***, and ****, indicating *p*-values < 0.05, 0.01, 0.001, and 0.0001, respectively.

## 5. Conclusions

In this study, we described an essential role for BAP31 in GAL-3/VEGF signaling, and our focus was primarily on establishing the angiogenic potential of BAP31. In conclusion, our findings indicate that BAP31 expression plays an important role in determining cellular modifications and the levels of cell migration, and possibly vascularization of neuroblastoma cancer, but the fact that BAP31 may have prognostic implications for neuroblastoma patients is an avenue yet to be researched. However, this study revealed key factors implicating BAP31-regulated neuroblastoma angiogenesis. Our results offer a foundation for further detailed investigations and propose that BAP31 may ultimately be a target for agents designed to treat patients with vascularized neuroblastoma.

## Data Availability

The data that support the findings of this study are available from the corresponding author upon reasonable request.

## Supplementary Materials

The following supporting information can be downloaded at: https://www.mdpi.com/article/10.3390/ijms25052946/s1.
