# Supplementary material for: BAP31 Promotes Angiogenesis via Galectin-3 Upregulation in Neuroblastoma
Source: Int J Mol Sci. 2024 Mar 3;25(5):2946. doi: 10.3390/ijms25052946 (PMC10931962; doi:10.3390/ijms25052946)
Supplement: Supplementary file 1 [file ijms-25-02946-s001.zip › ijms-2847600-supplementary.pdf]

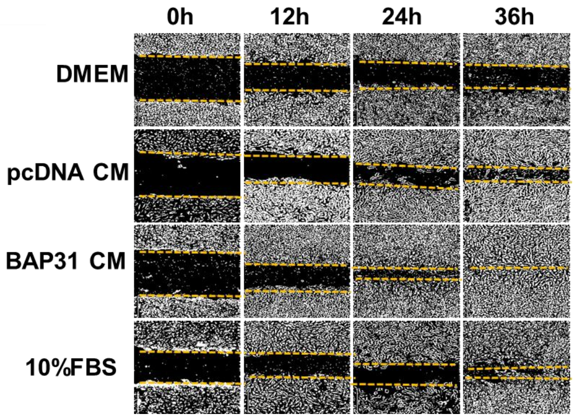

Figure S1 Representative photomicrographs of the wound area and closure over time in Human endothelial cells cultured in different media. The wound closure from the fastest to slowest was as follows: BAP31 CM, pcDNA CM, 10% FBS and DMEM (negative control).

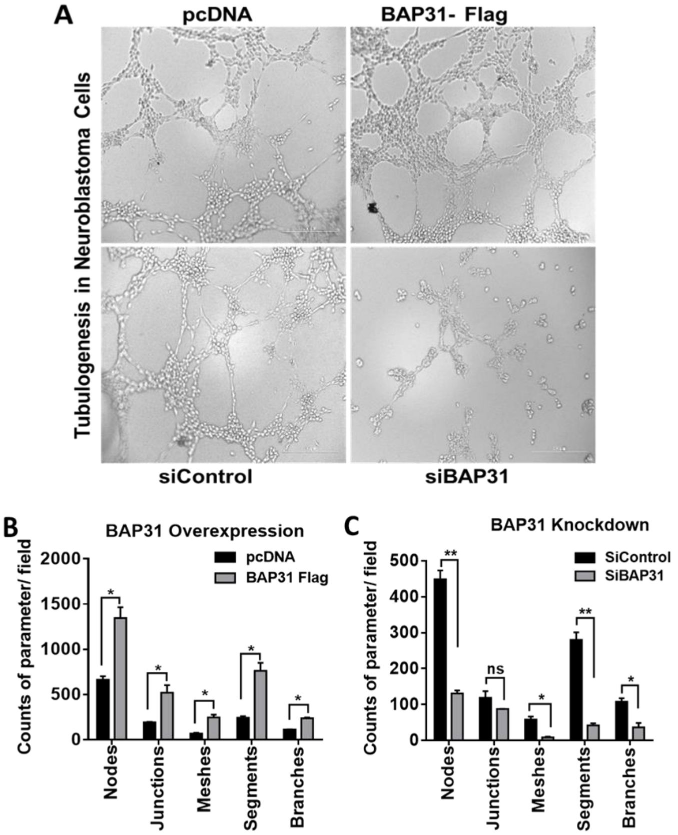

Figure S2 BAP31 modulates SH-SY5Y cell morphology towards an angiogenic phenotype. **(A)** Representative micrographs of vascular mimicry by SH-SY5Y cells with controls (pcDNA for BAP31 overexpression, and siControl for BAP31 knockdown). **(B)** BAP31 overexpression tube formation parameters. **(C)** BAP31 knockdown tube formation parameters. Statistical significance was determined by a Student's t-test (*$p < 0.05$, **$p < 0.01$, ***$p < 0.001$ and **** $p < 0.0001$).

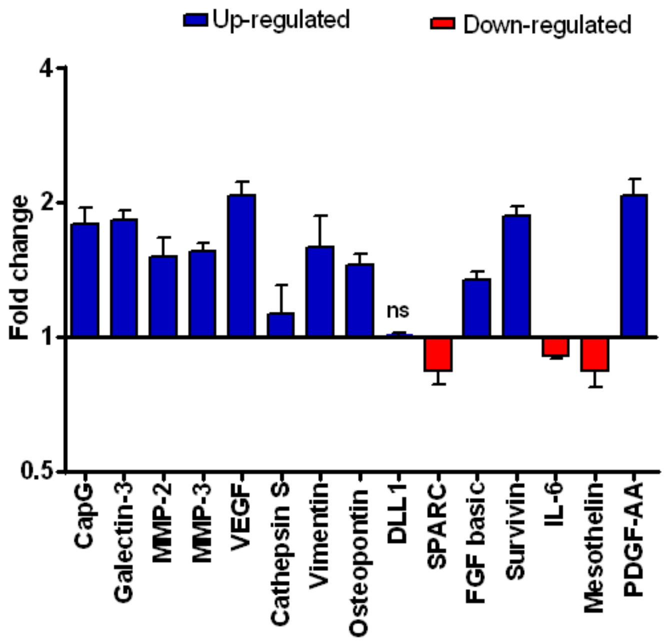

Figure S3 Fold change of selected pro-angiogenic and anti-angiogenic factors from an oncology array assay following BAP31 overexpression in the SH-SY5Y cell line. The fold diffrences where as folows: CapG (1.7), Galectin-3 (1.8), MMP-2 (1.5), MMP-3 (1.6), VEGF (2.1), Cathepsin S (1.1), Vimentin (1.6), Osteopontin (1.5), DLL1 (1.0), SPARC (0.8), FGF basic (1.3), Survivin (1.9), IL-6 (0.9), Mesothelin (0.8), PDGF-AA (2.1).
